# Addressing Risks: Mental Health, Work-Related Stress, and Occupational Disease Management to Enhance Well-Being 2019

**DOI:** 10.1155/2020/1863153

**Published:** 2020-06-19

**Authors:** Gabriele Giorgi, Jose M. Leon-Perez, Silvia Pignata, Gabriela Topa, Nicola Mucci

**Affiliations:** ^1^Department of Human Sciences, European University of Rome, Via Degli Aldobrandeschi, 190, 00163 Rome, Italy; ^2^Department of Social Psychology, University of Seville, Camilo Jose Cela s/n, 41018 Sevilla, Spain; ^3^Aviation Faculty School of Engineering, University of South Australia, Adelaide, SA, Australia; ^4^Department of Social and Organizational Psychology, National Distance Education University (UNED), Madrid 28040, Spain; ^5^Department of Experimental and Clinical Medicine, University of Florence, Largo Piero Palagi 1, 50139 Florence, Italy

Biomed Research International, section Public Health, decided to transform the Special Issue “Addressing Risks: Mental Health, Work-Related Stress, and Occupational Disease Management to Enhance Well-Being” published in 2018 [1] into a permanent special issue.

The importance of a contextualized health approach with a focus on organizational environments is becoming more strategic than ever due to COVID-19 and the ensuing difficult situation that employees are experiencing worldwide. The prevention of workers' mental health problems is complex and multidimensional, and it is not always possible to protect the person by analyzing personality, psychopathology, and psychiatric syndromes.

Accordingly, a peer review process involving international experts, with 21 papers accepted in this special issue, considered the important concept of work contextualized health. From this perspective, this special issue had the power to establish a dialogue between the multiple disciplines completing the majority of research on mental health constructs within clinical, neuroscientific, and psychiatric contexts, which usually led to a person-centered analysis or research conducted in artificial laboratory settings. As stated by Giorgi et al. [1], trauma and diseases related to stress and mental health that originate in the workplace may have a different pattern of development or require an organization-centered treatment approach, including field and intervention studies.

In addition, this special issue followed the United Nations agenda for developing 17 sustainable goals by 2030 [2] and tried to contribute to two of these goals: (1) promoting well-being at all ages, including mental health, and (2) promoting safe and secure working environments to create a decent work for all. In doing so, this special issue assumed that addressing such goals required an interdisciplinary approach involving scientific fields ranging from occupational medicine to organizational psychology.

Regarding the promotion of well-being at all ages, G. Giorgi et al. concluded in their narrative review that stress management strategies at work need to include “aging” as a crucial variable to tailor interventions and prevent workers' cognitive impairment processes. Also, M. Ziarko et al. pointed out the necessity to consider the health and well-being of workers with chronic disease. In particular, their paper analyzed the mental health consequences of the type of treatment received by 85 participants affected by rheumatoid arthritis and confirmed the assumption that pain intensity, coping strategies, and ego resiliency depend on the severity of their levels of anxiety and depression.

Similarly, two papers emphasized the need to consider all agents involved in an organization, including students, as targets of safety and well-being measures. K. Gerreth et al. analyzed the anxiety of dental students during their first clinical class involving performing a prophylactic procedure in a pediatric patient. Their results indicated that more than 51% of students reported high levels of anxiety. These findings emphasize the need for students to be trained to deal with stress as a part of their academic curriculum. In addition, K. Frömel et al. explored whether students reporting academic stressors differ in physical activity after school compared to those students that did not report being exposed to academic stressors. Although their hypotheses were not supported, it seems that gender should be taken into account when promoting physical activity to reduce stress: girls in the academic stressor group walked more (steps/hour measured with accelerometers) than girls in the nonacademic stressor group. Also, their study is a good example on how new devices such as accelerometers can be used to collect information in occupational health and safety research.

With regard to promoting safe and secure working environments to create a decent work for all, some papers published in this special issue introduce advances in measuring psychosocial risk factors, mental health, and work-related issues. For example, N. Tao et al. conducted a study in which they analyzed the relationship between occupational stress and secretory immunoglobulin A (sIgA) in a sample of 625 military recruits during their basic military training period. As expected, sIgA measured in saliva and quantified by enzyme-linked immunosorbent assay presented higher levels in the high occupational stress group than that in the low stress group. Furthermore, the salivary sIgA level was also associated with perceived personal strain.

Another work using innovative approaches to assess psychosocial risks and their consequences is the paper by J. R. López-García et al. They proposed using Bayesian networks to determine the probability of an occupational accident in a certain productive sector depending on the relationship between ergonomic and psychosocial factors. They used data from a national survey of working conditions in Spain (*n* = 8,892) to illustrate their approach. Their results suggest that ergonomic risks associated with physical strains and a lack of job satisfaction are associated with a higher probability of being involved in an occupational accident.

In contrast to these new approaches, traditional approaches to conduct psychosocial risk assessments are based on self-rated scales. In that sense, it is important to validate well-known scales to facilitate cross-cultural comparisons. This is the case of the study conducted by A. S. N. Isha et al. who validated the Copenhagen Psychosocial Work Environment Questionnaire (COPSOQ) in Malaysia. They also proposed the inclusion of physiological measures (blood pressure and body mass index) to monitor workers' health.

Similarly, V. Katsari et al. validated the Jefferson Scale of Patient Perception of Physician Empathy in Greece, which may be useful for monitoring both physicians' health and the quality of service that they provide. A noteworthy aspect of this study was the comparison between self-rated empathy and their patients' ratings. A similar approach was followed by I. Schneider et al. in their research on the degree of agreement between self-rated and observer-rated occupational psychosocial risks. They compared the ratings of workers and occupational safety and health committees to occupational psychosocial risks measured with the same instrument (*n* = 669). Their findings showed that observer ratings and self-ratings provided comparable results. Therefore, they concluded that (a) the observer rating approach is especially suitable for small-to-medium enterprises that do not have access to a large anonymous survey assessment and (b) aggregation of item means at the group level is justified because their results showed a reasonable agreement and excellent reliability in workers' self-ratings, and therefore, the self-rating approach can be very useful for large enterprises.

Another way to improve existing scales to measure psychosocial risks at work is to add relevant dimensions that are associated with employees' health and well-being. In that sense, the work by K. Kowalczuk et al. attempted to identify the most arduous and frequently occurring burdens in nursing workplaces. They found that ward type predicted the level of work arduousness beyond other factors such as age or gender, suggesting that trauma and diseases related to stress and mental health that originate in the workplace may have a different pattern of development or require an organization-centered treatment approach that complements the person-centered approach derived from research conducted in clinical and psychiatric contexts.

In a similar vein, M. Martini et al. highlighted the importance of including both demands and support derived from interactions with students when conducting psychosocial risk assessments in higher education. With a sample of 550 professors from a large public university, their results revealed that relationships with students can play a crucial role in how academics experience emotional exhaustion and engagement at work. Also, findings from the study conducted by M. del Mar Molero Jurado et al. in the education sector reaffirm that burnout is a pivotal psychosocial risk that requires prevention within the sector. They proposed that measures to prevent burnout need to consider the educational context when implementing preventative actions both at the individual (i.e., increasing self-efficacy) and organizational level (i.e., improving the education system). Moreover, organizational level measures should include the promotion of healthy behaviors as emphasized by research on public health initiatives to prevent noncommunicable diseases [3]. This is clearly exemplified in the study by A. Habib et al. who analyzed the risk factors of noncommunicable diseases in a sample from Saudi Arabia (*N* = 1,070). Their findings revealed the need to promote healthy behaviors as a suitable public health strategy to reduce noncommunicable diseases such as cardiovascular disease or diabetes.

In addition to these potential measures to promote health and well-being, the literature has indicated that active coping and recovery from work are crucial to avoid stress-related problems [4, 5]. In that sense, the paper by Y. Hsu et al. reported that working more hours was associated with higher levels of occupational stress, which was related to lower levels of work-family balance and job satisfaction. They found that perceived control over time plays a protective role because it was associated with increased recovery-related self-efficacy. In addition, a focus on coping strategies by X. Wang et al. revealed that depressive symptoms in military institutions is a matter that needs to be considered, as they found that the relationship between coping (i.e., hardiness) and depressive symptoms is mediated by motivational dispositions.

Addressing psychosocial risks and introducing preventive measures at work are equally important as identifying who is exposed to the risks and what are the potential negative consequences on employees' health and well-being. First, A. Przystanska et al. explored the psychosocial predictors of bruxism. They concluded that perceived stress is a crucial somatic factor in the occurrence and maintenance of awake bruxism. Second, K. Golonka et al. went beyond the usual negative effects of burnout and explored potential brain activity differences between burned-out and nonburned workers (control group). Their results suggest that participants in the burnout group showed cortical hyperactivity, which results in reduced alpha power compared to participants in the control group. Finally, T. Mitake et al. analyzed the stigma related to mental illness in the workplace, such as the psychological consequences derived from burnout. This relationship is important to examine because being stigmatized at work due to mental illness can result in experiencing discriminative behaviors.

Following the abovementioned findings, another factor that deserves special attention to create a decent work for all is the promotion of working environments free from discrimination and violence, including sexual and psychological violence (i.e., sexual harassment or workplace bullying). The paper by S. A. Jahnke et al. addressed the prevalence of chronic work discrimination and the harassment of women firefighters (*n* = 1,773) and its psychosocial consequences. Their results revealed that a considerable percentage of women firefighters reported that they had experienced verbal harassment (37.5%) and unwanted sexual advances (37.4%) in their fire service work. Furthermore, this discrimination and harassment at work were related to increased alcohol consumption and mental health problems, including depressive symptoms, anxiety, and posttraumatic stress symptoms. Similarly, S. Berlanda et al. analyzed the experiences of violence (emotional, physical, and sexual) perpetrated by patients and visitors against healthcare professionals working in emergency units. They found that greater age and higher scores in secure attachment are associated with reduced experience of emotional violence from patients and visitors, and the relationship between secure attachment and the amount of patient-and-visitor-perpetrated emotional violence experienced is mediated by levels of job satisfaction.

Finally, with regard to the ongoing situation and the economic crisis caused by the COVID-19 pandemic, employee welfare and social support may not be the current priorities for companies as they attempt to maintain their survival by staff layoffs and budget reductions [6, 7]. Moreover, studies have shown that turbulent economic periods, in which job uncertainty is the norm, create a fertile soil for the increase of violence at work and stress-related mental health problems [6, 8]. In this regard, S. De Sio et al. studied the role of job insecurity in the perception of psychosocial risks at work in a sample of 338 administrative technical workers and found that workers with temporary contracts perceived higher exposure to psychosocial risks at work than their colleagues with permanent contracts.

The contributions to this special issue highlight the essential need to consider organizational practices and culture in the management of mental health problems linked to the workplace as organizational causes are often more harmful than individual antecedents. Raising awareness of the organization's intervention politics, of organization-worker health relationships at work, and of an organizational science of mental health appears necessary. Overall, the manuscripts included in this special issue reported the perspectives of 123 authors, reflecting a valuable cross-cultural point of view on health prevention and promotion.

In conclusion, we would like to share a reflection on what we have seen during the COVID-19 pandemic as workers' mental health still represents a point of fragility of the systems-countries, where an overly medicalized and pathologizing model of mental health risks hiding not only organizational causes and responsibilities creating an image of stigmatized workers but also hiding potential and successful organizational interventions in prevention, safety, and health areas. There is therefore a disharmonious relationship between business and health while, as strongly supported by the Business@Health laboratory of the European University of Roma, there is no business without employee health, and in the same way, employee health becomes business.

*Gabriele Giorgi* *Jose M. Leon-Perez* *Silvia Pignata* *Gabriela Topa* *Nicola Mucci*
